# Supplementary material for: Protein Kinase A Distribution in Meningioma
Source: Cancers (Basel). 2019 Oct 29;11(11):1686. doi: 10.3390/cancers11111686 (PMC6895821; doi:10.3390/cancers11111686)
Supplement: Supplementary file 1 [file cancers-11-01686-s001.pdf]

Supplemental Material

Protein Kinase A Distribution in Meningioma

Antonio Caretta, Luca Denaro, Domenico D'Avella, Carla Mucignat-Caretta

Table 1. Cases.

NUMBER	SEX	AGE at surgery	DIAGNOSIS	Notes
1	F	78	Fibrous meningioma	*Some sclerosis areas. Grade I**
2	F	23	Meningioma	*Some sclerosis areas. Grade I**
3	F	66	Meningothelial meningioma	Grade I WHO
4	F	62	Meningothelial meningioma	Grade I WHO
5	F	77	Atypical meningioma	*Grade II**
6	F	70	Transitional meningioma with psammomatous areas	Grade I WHO
7	F	52	Meningioma	*Grade I**
8	M	60	Atypical meningioma	Grade II WHO. Recidiva
9	F	2	Meningioma	*Grade I**
10	F	43	Anaplastic meningioma	Grade III WHO
11	M	77	Atypical meningioma	Grade II WHO
12	M	76	Meningothelial meningioma	Grade I WHO
13	F	52	Meningioma	*Grade I**

In *Italics* *: grading deduced from diagnosis, when not indicated by the pathologist.

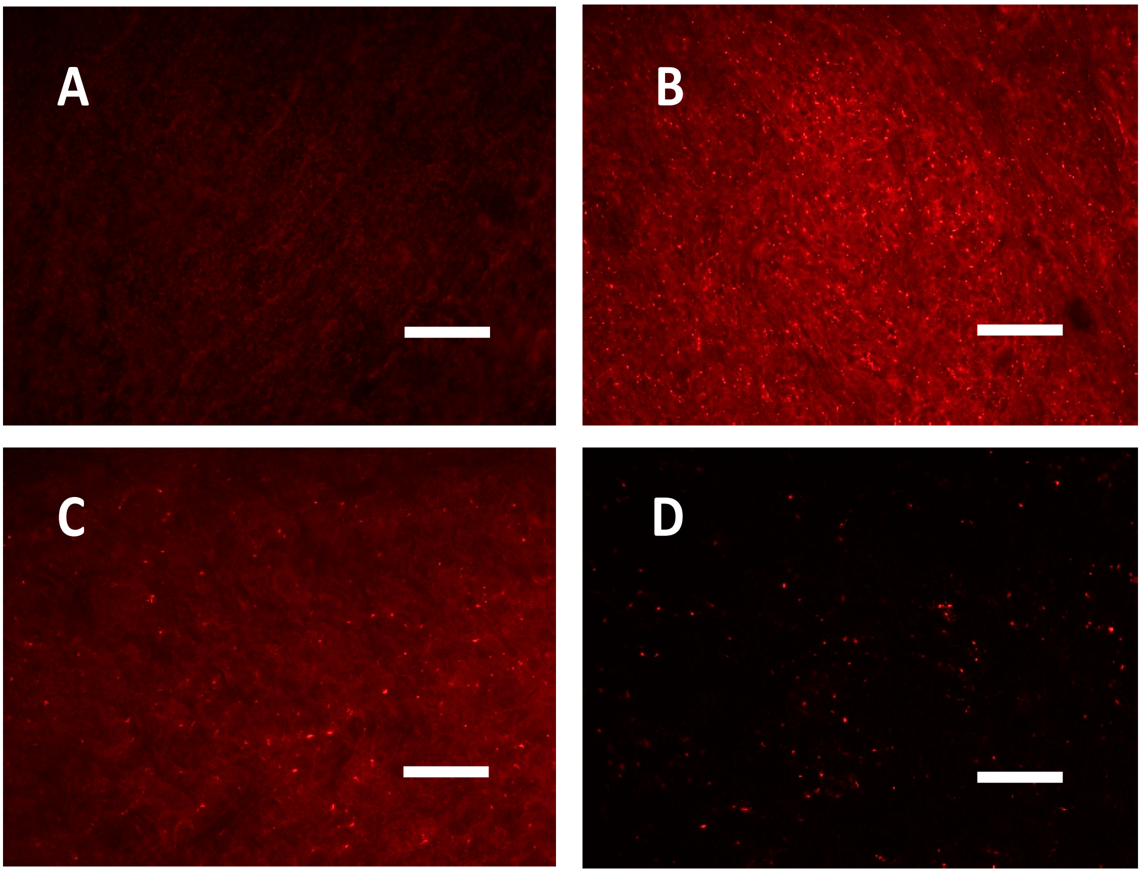

Figure S1. Optimization of fixation protocols. Meningioma case 1 serial sections. PKA catalytic subunit immunofluorescence was revealed with AlexaFluor594 secondary antibody, see main text for details. **A**: Mild fixation: 1 minute in formalin at 37°C. **B**: Mild fixation followed by permeabilization: 1′ in formalin at 37°C, then Triton X-100 2% in PBS for 30′ at room temperature. **C**: formalin 1 hour at room temperature, followed by Triton X-100 2% in PBS for 30′. **D**: permeabilization in Triton X-100 2% in PBS for 30′, followed by fixation in formalin 1′ at 37°C. 40X objective (**A–D**), exposure time: 1′′28 (**A**, **C**, **D**) or 0.80′′ (**B**). Bar = 25 m. Unaltered images.

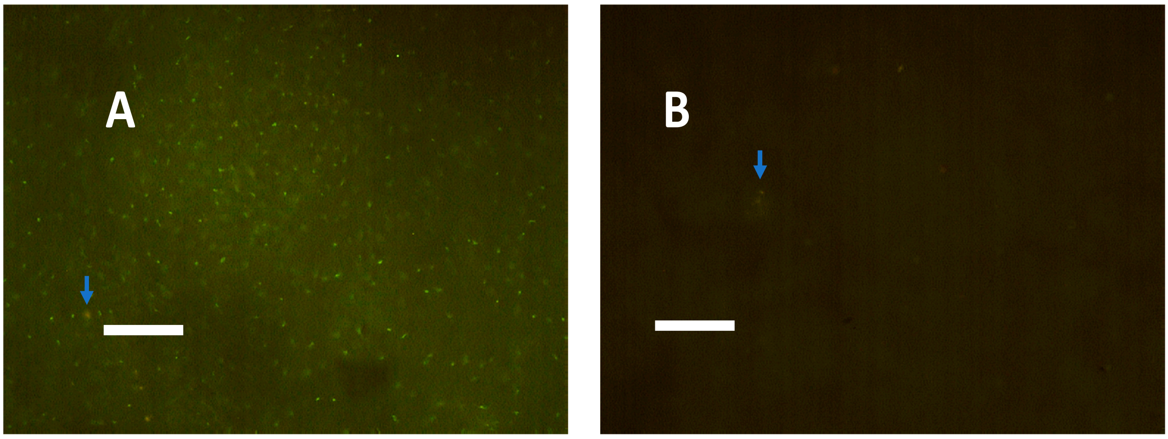

Figure S2. Fluorescent cAMP binding to meningioma case 3. Mild fixation + permeabilization (1′ in formalin at 37°C, then Triton X-100 2% for 30′ at room temperature). **A**: slides were incubated with SAF-cAMP 300 nM for 10 minutes at room temperature, then observed at the microscope. **B**: slides were incubated with SAF-cAMP 300 nM + 8Br-cAMP 10 mM, for 10 minutes at room temperature, then observed at the microscope. Autofluorescence can be seen as blurred reddish areas (one example in each image indicated by blue arrows). Only in A the bright green fluorescence of SAF-cAMP is present. 40X objective, exposure time: 1′′28. Luminosity increased by 30% to show also barely detectable autofluorescence. Bar = 25 m.

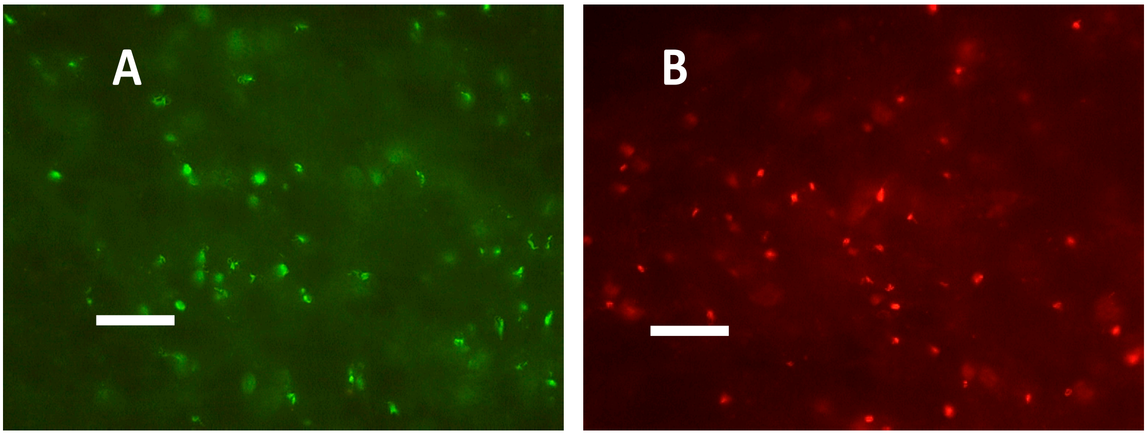

Figure S3. Fluorescent cAMP binding to meningioma case 5. Unfixed tissue. **A**: slide was incubated with Alexa 488-cAMP 100 nM for 10 minutes at room temperature, then observed at the microscope. **B**: slide was incubated with Alexa 555-cAMP 100 nM, for 10 minutes at room temperature, then observed at the microscope. 100X objective, exposure time: 1’’64. Bar = 10 m.

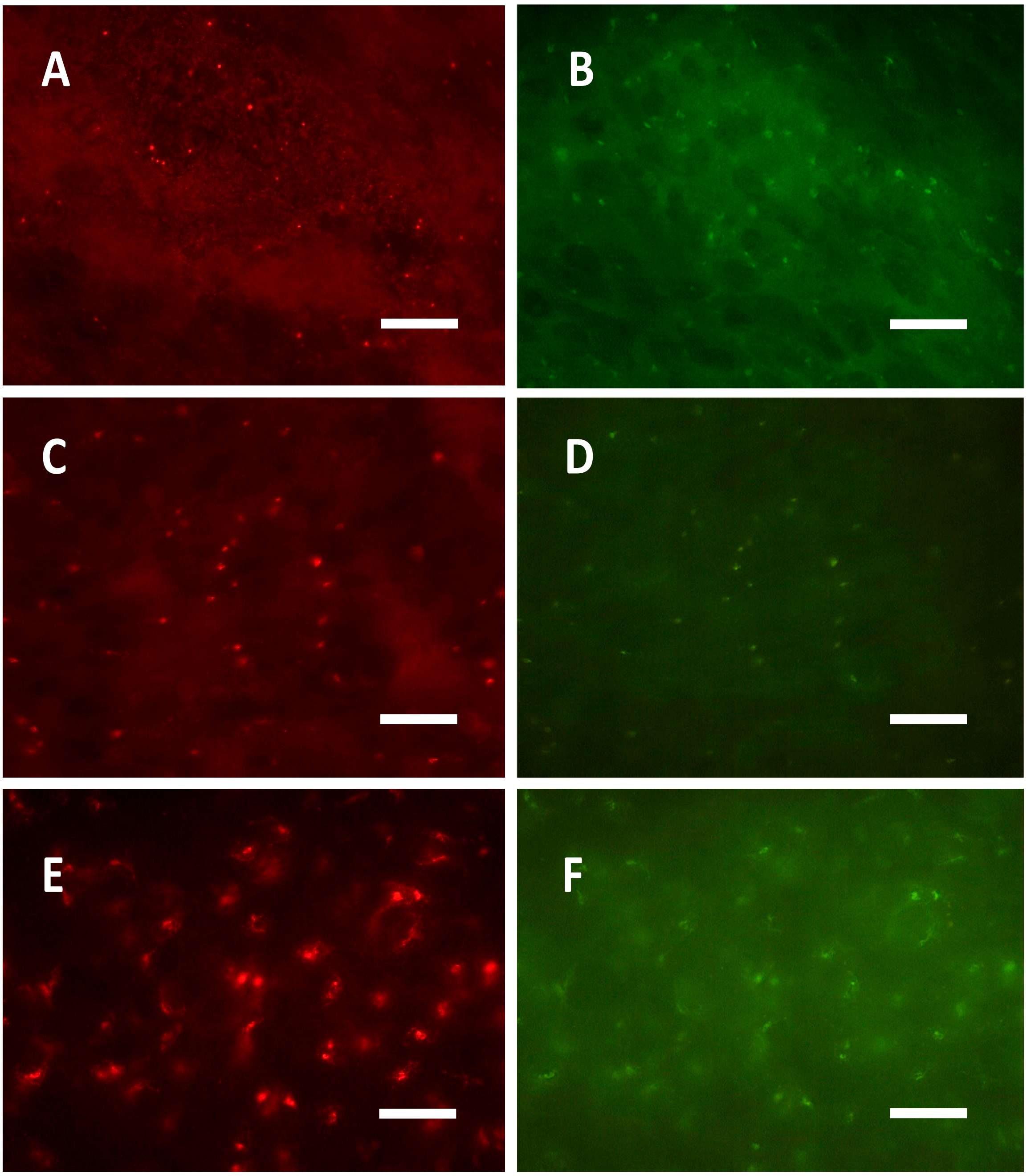

Figure S4. Original single-channel images, relative to Figure 3 (see Main Text). Fluorescent cAMP binding coupled to immunofluorescence. **A and B**: meningioma case 5, same field, slide was incubated with anti-PKA RI (**A**, red) then with SAF-cAMP 300 nM (**B**, green) for 10 minutes: no colocalization is present. **C and D**: meningioma case 5, same field, slide was incubated with anti-PKA RII (**C**, red) then with SAF-cAMP 300 nM (**D**, green) for 10 minutes at room temperature, apparently labelling the same structures. **E and F**: meningioma case 3, same field, slide was incubated with anti-PKA catalytic subunit (**E**, red) then with Alexa 488-cAMP 100 nM (**F**, green) for 10 minutes at room temperature, apparently labelling the same structures. Mild fixation coupled to permeabilization, 100X objective. Bar = 10 m. Unaltered images.

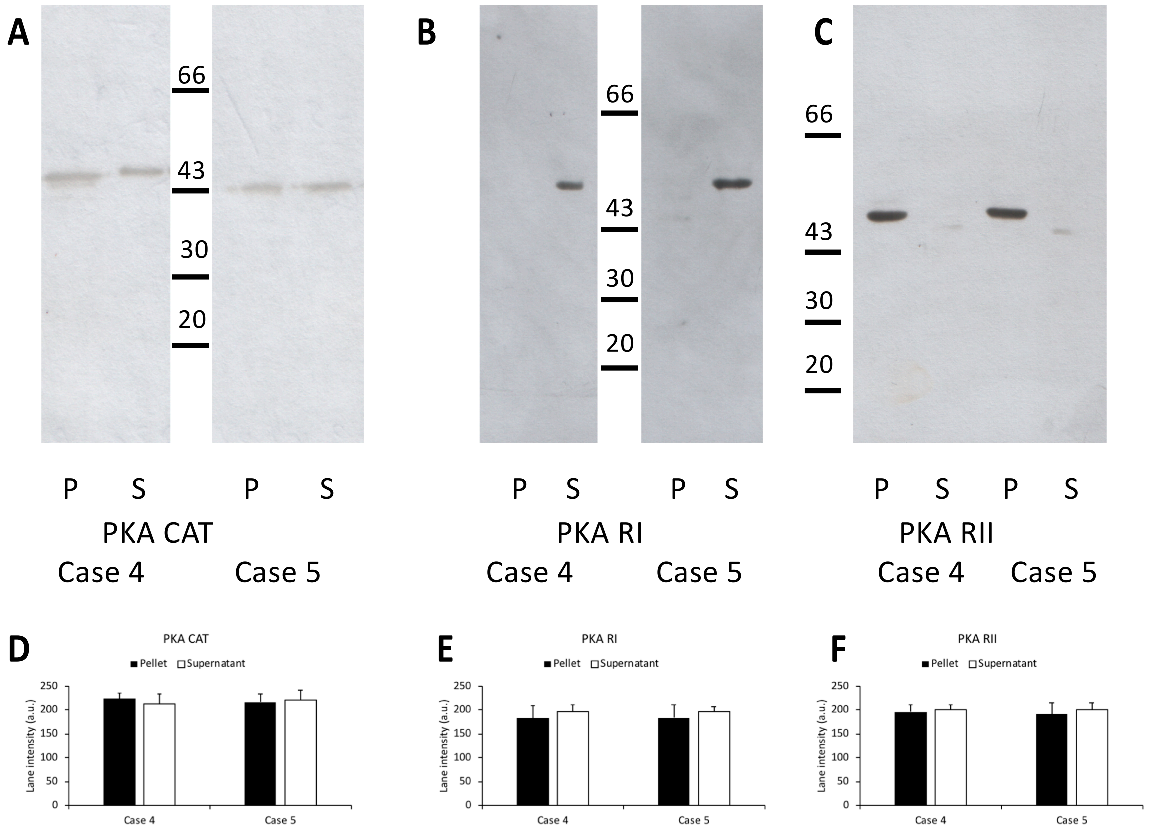

Figure S5. Western blot analysis was only possible for two meningioma patients (case 4 and 5) of the present series. **A–C**: unmodified images; numbers indicate apparent molecular mass. **A**: PKA catalytic (PKA CAT) subunit expression in insoluble (pellet, P) and soluble (supernatant, S) fractions in cases 4 and 5. **B**: PKA RI (PKA RI) subunit expression in insoluble and soluble fractions in cases 4 and 5. **C:** PKA RII (PKA RII) subunit expression in insoluble and soluble fractions in cases 4 and 5. **D**: quantification of lane loading, referred to **A**. **E**: quantification of lane loading, referred to **B**. **F**: quantification of lane loading, referred to **C**.

Western Blot Methods

All chemicals were from Sigma, Milan, Italy, unless otherwise stated. Samples were homogenized in 40x volume PBS (140 mM NaCl, phosphate buffer 10 mM pH = 7.4, ethylenediaminetetraacetic acid 1 mM), 2 mM phenylmethanesulfonylfluoride, protease inhibitor cocktail (Roche, Mannheim, Germany) and 10 mg/ml soy-bean trypsin inhibitor. The supernatants were removed, the pellets were washed thrice and resuspended in PBS. After protein quantification (Bradford assay, BioRad, Richmond, CA), five microliters of the properly diluted supernatant and pellet were loaded on 12% sodium dodecyl-sulfate (SDS) polyacrylamide gel (BioRad, Richmond, CA), that were subsequently blotted onto nitrocellulose membrane (Whatman International, Maidstone, England), blocked in 2% bovine serum albumin in Tris buffer 20 mM, NaCl 500 mM, pH = 7.5 and incubated overnight with either anti-PKA catalytic, RI or RII subunit (Santa Cruz Biotechnology, see Materials and Methods, 1:5000). The secondary antibody conjugated with horseradish peroxidase (Sigma, 1:10000) was incubated for four hours and developed with chemiluminescence (Advanced ECL, Amersham, Milan, Italy). Since the use of different housekeeping proteins has been questioned (Barber et al., 2005; Dittmer and Dittmer, 2006; Zhong and Simons, 1999), the staining of all protein bands in each lane was used as loading control, according to Welinder et al. (2011). Western blots were acquired linearly at 1200 dpi using the same parameters for each film and handled with GraphicConverter9.

References

1. Barber, R.; Harmer, D.; Coleman, R.; Clark B. GAPDH as a housekeeping gene: analysis of GAPDH mRNA expression in a panel of 72 human tissues. *Physiol. Genomics* **2005**, *21*, 389–395.
2. Dittmer, A.; Dittmer, J. Beta-actin is not a reliable loading control in Western blot analysis. *Electrophoresis* **2006**, *27*, 2844–2845.
3. Welinder, C.; Ekblad, L. Coomassie staining as loading control in Western blot analysis. *J. Proteome Res.* **2011**, *10*, 1416–1419.
4. Zhong, H.; Simons, J. Direct comparison of GAPDH, beta-actin, cyclophilin, and 28S rRNA as internal standards for quantifying RNA levels under hypoxia. *Biochem. Biophys. Res. Commun.* **1999**, *259*, 523–526.
